# Screening and cDNA Cloning of Kv1 Potassium Channel Toxins in Sea Anemones

**DOI:** 10.3390/md8122893

**Published:** 2010-12-02

**Authors:** Yoshikazu Yamaguchi, Yuichi Hasegawa, Tomohiro Honma, Yuji Nagashima, Kazuo Shiomi

**Affiliations:** 1 Department of Food Science and Technology, Tokyo University of Marine Science and Technology, Konan-4, Minato-ku, Tokyo 108-8477, Japan; 2 Department of Food Nutrition, Tokai University Junior College, Miyamae-cho, Aoi-ku, Shizuoka 420-8511, Japan

**Keywords:** cDNA cloning, potassium channel toxin, screening, sea anemone

## Abstract

When 21 species of sea anemones were screened for Kv1 potassium channel toxins by competitive inhibition of the binding of ^125^I-α-dendrotoxin to rat synaptosomal membranes, 11 species (two species of Actiniidae, one species of Hormathiidae, five species of Stichodactylidae and three species of Thalassianthidae) were found to be positive. Furthermore, full-length cDNAs encoding type 1 potassium channel toxins from three species of Stichodactylidae and three species of Thalassianthidae were cloned by a combination of RT-PCR, 3′RACE and 5′RACE. The precursors of these six toxins are commonly composed of signal peptide, propart and mature peptide portions. As for the mature peptide (35 amino acid residues), the six toxins share more than 90% sequence identities with one another and with κ_1.3_-SHTX-She1a (Shk) from *Stichodactyla helianthus* but only 34–63% identities with the other type 1 potassium channel toxins.

## 1. Introduction

Sea anemones contain various classes of peptide toxins, especially those acting on sodium or potassium channels [1–3]. The most extensively studied peptide toxins are sodium channel toxins that delay channel inactivation during the depolarization process by binding to the receptor site 3 [1,4–8]. In the mid-1990s, peptides blocking potassium channels were discovered in some sea anemones. Interestingly, potassium channel toxins act synergistically with sodium channel toxins to delay the repolarization phase of action potentials. A total of 15 potassium channel toxins isolated so far can be structurally classified into four types [9]: type 1 toxins (35–37 amino acid residues) include κ_1.3_-ATTX-Aeq1a (AeK; toxin names are described in this paper based on the nomenclature proposed by King *et al*. [10], with their original names at the first appearance) from *Actinia equina* [11], κ_1.3_-ATTX-Aer1a (AETX K) from *Anemonia erythraea* [12], κ_1.3_-ATTX-As1a (AsKS or kaliseptine) from *Anemonia sulcata* [13], κ_1.3_-ATTX-Bg1a (BgK) from *Bunodosoma granulifera* [14], κ_1.3_-SHTX-Hm1a (HmK) from *Heteractis magnifica* [15] and κ_1.3_-SHTX-She1a (ShK) from *Stichodactyla helianthus* [16]; type 2 toxins (58–62 amino acid residues), which are homologous with Kunitz-type protease inhibitors, include κ_1.3_-ATTX-As2a-c (AsKC1-3 or kalicludines 1–3) from *A. sulcata* [13] and κ_1.3_-SHTX-Sha2a (SHTX III) from *Stichodactyla haddoni* [17]; type 3 toxins (42 or 43 amino acid residues) include κ_3.4_-ATTX-As1a and b (BDS-I and II) from *A. sulcata* [18] and κ-ATTX-Ael1a (APETx1) from *Anthopleura elegantissima* [19,20]; and type 4 toxins (28 amino acid residues) include κ_1.3_-SHTX-Sha3a and b (SHTX I and II) from *S. haddoni* [17]. The majority of the above potassium channel toxins are blockers of Kv1 potassium channels, except for the type 3 toxins (κ_3.4_-ATTX-As1a and b which modulate Kv3.4 potassium channels [18] and κ-ATTX-Ael1a which modulates human *ether-a-go-go*-related gene potassium channels [19,20]). In addition, Kv1 potassium channel toxins, although not isolated, have also been detected in three species of sea anemones (*Actinia bermudensis*, *Bunodosoma cangicum* and *Stichodactyla mertensii*) [21]. Thus, Kv1 potassium channel toxins are expected to still exist in sea anemones that have not been studied.

It should be noted that all the sea anemones that have been shown to contain potassium channel toxins, including Kv1 channel toxins, belong to either the family Actiniidae or the family Stichodactylidae. Our previous screening failed to detect Kv1 potassium channel toxins in any of the five species of the families differing from the above two families [22]. In this study, therefore, screening for Kv1 potassium channel toxins, which was based on the competitive inhibition of the binding of radiolabeled α-dendrotoxin (Kv1 potassium channel toxin from the green mamba *Dendroaspis angusticeps* [23]) to rat synaptosomal membranes, was further extended to 21 species of sea anemones in seven families. Furthermore, molecular cloning was attempted to elucidate the primary structures of type 1 potassium channel toxins, for which degenerate primers could be designed from the known nucleotide sequences of the cDNAs encoding κ_1.3_-ATTX-Aer1a [12] and κ_1.3_-SHTX-Hm1a [15].

## 2. Results and Discussion

### 2.1. Screening of potassium channel toxins

**Figure 1:**
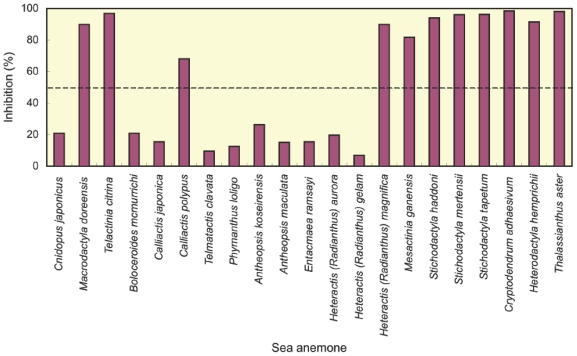
Inhibition of the binding of ^125^I-α-dendrotoxin to rat synaptosomal membranes by crude extracts from 21 species of sea anemones. Each datum is a mean of two determinations.

Crude extracts from 21 species of sea anemones were examined for Kv1 potassium channel toxicity by competitive inhibition experiments. As shown in Figure 1, inhibition of the binding of ^125^I-α-dendrotoxin to rat synaptosomal membranes was observed in all species with varied potencies. The species with only weak inhibitory activity were considered to be hardly selected as samples in future study on potassium channel toxins. In this study, therefore, the following 11 species showing more than 50% inhibition were judged to be substantially positive: two species (*Macrodactyla doreensis* and *Telactinia citrina*) of the family Actiniidae, one species (*Calliactis polypus*) of the family Hormathiidae, five species (*Heteractis magnifica*, *Mesactinia ganensis*, *Stichodactyla haddoni*, *Stichodactyla mertensii* and *Stichodactyla tapetum*) of the family Stichodactylidae and three species (*Cryptodendrum adhaesivum*, *Heterodactyla hemprichii* and *Thalassianthus aster*) of the family Thalassianthidae. One Kv1 potassium channel toxin (κ_1.3_-SHTX-Hm1a) has already been isolated from *H. magnifica* [15] and three Kv1 potassium channel toxins (κ_1.3_-SHTX-Sha2a, κ_1.3_-SHTX-Sha3a and κ_1.3_-SHTX-Sha3b) from *S. haddoni* [17]. Furthermore, previous screening has established the occurrence of Kv1 potassium channel toxins in *S. mertensii* [21]. The remaining eight species were first demonstrated to be positive in this study. So far, Kv1 potassium channel toxins have not been found in any species other than those belonging to the family Actiniidae or Stichodactylidae. In view of this, our screening data are of particular value in showing the occurrence of Kv1 potassium channel toxins in one species of Hormathiidae and three species of Thalassianthidae.

Based on our results and previous data, distribution of Kv1 potassium channel toxins in sea anemones is summarized in Table 1. Of the 44 species examined, 18 species belonging to four families (Actiniidae, Hormathiidae, Stichodactylidae and Thalassianthidae) contain Kv1 potassium channel toxins. In general, sodium channel toxins are lethal to crustaceans. On the other hand, potassium channel toxins are not lethal to crustaceans, although some of them, such as three toxins (κ_1.3_-SHTX-Sha2a, κ_1.3_-SHTX-Sha3a and κ_1.3_-SHTX-Sha3b) from *S. haddoni* [17], are paralytic. To our experience, crude extracts from various sea anemones are lethal to freshwater crabs (*Potamon dehaani*) without exception, indicating a ubiquitous distribution of sodium channel toxins in sea anemones. Therefore, the distribution of Kv1 potassium channel toxins in sea anemones is considerably wide but seems to be narrower than that of sodium channel toxins.

Five species of the genus *Stichodactyla* in the family Stichodactylidae are all positive, suggesting the common occurrence of Kv1 potassium channel toxins in this genus. Similarly, three species of the family Thalassianthidae are all positive, although they are classified into different genera. It is likely that members of Thalassianthidae commonly contain Kv1 potassium channel toxins. In addition, Kv1 potassium channel toxins might be widely distributed in members of the three genera (*Actinia*, *Anemonia* and *Bunodosoma*) in the family Actiniidae, although only two species in each genus have been tested and found to be positive. Further screening experiments using much more species are needed to confirm the relationships between the occurrence of Kv1 potassium channel toxins and the taxonomical position of sea anemones.

### 2.2. Cloning of cDNAs encoding type 1 potassium channel toxins

RT-PCR using a pair of degenerate primers (RT-f and RT-r; refer to Table 2 for the nucleotide sequences of the primers) was performed for the following eight species: the six positive species (*M. doreensis*, *S. haddoni*, *S. mertensii*, *C. adhaesivum*, *H. hemprichii* and *T. aster*) found in this study, *A. equina* previously shown to have a potassium channel toxin (κ_1.3_-ATTX-Aeq1a) [11] and *S. gigantea* (a member of the genus *Stichodactyla*) strongly assumed to have a potassium channel toxin. Amplified products were obtained for three *Stichodactyla* species (*S. gigantea*, *S. haddoni* and *S. mertensii*) of the family Stichodactylidae and three species (*C. adhaesivum*, *H. hemprichii* and *T. aster*) of the family Thalassianthidae but not for two species (*A. equina* and *M. doreensis*) of the family Actiniidae. As expected, approximately 150 bp products were amplified for *S. haddoni*, *C. adhaesivum* and *H. hemprichii*. However, longer products (approximately 350 bp), corresponding to the region between the forward primer (RT-f) position and the 3′-end, were amplified for *S. gigantea*, *S. mertensii* and *T. aster*, probably because the degenerate reverse primer (RT-r) did not anneal to the template. Therefore, 3′RACE to analyze the nucleotide sequences of the 3′-terminal regions was carried out only for *S. haddoni*, *C. adhaesivum* and *H. hemprichii*. Finally, nucleotide sequences of the 5′-terminal regions were determined by 5′RACE.

**Figure 2:**
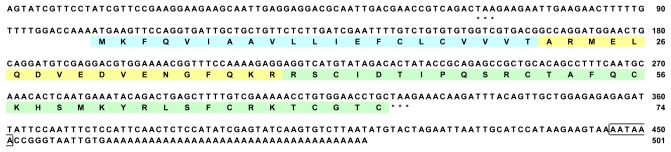
Nucleotide sequence of the cDNA encoding κ_1.3_-SHTX-Sg1a. Deduced amino acid sequence is aligned below the nucleotide sequence. Nucleotide and amino acid numbers are shown on the right. In-frame stop codons (TAA) are shown by asterisks. A poly(A) signal is boxed. Putative signal peptide, propart and mature peptide are shaded with light blue, yellow and light green, respectively.

After subcloning each PCR product into the pT7Blue T-vector, at least three clones were analyzed for nucleotide sequence. For each PCR product, there was no difference in nucleotide sequence among the clones analyzed, suggesting that isoforms of the cloned toxin, if present, are trace in the six species. The determined nucleotide sequences of the full-length cDNAs coding for six type 1 potassium channel toxins (named κ_1.3_-SHTX-Sg1a, κ_1.3_-SHTX-Sha1a, κ_1.3_-SHTX-Sm1a, κ_1.3_-TLTX-Ca1a, κ_1.3_-TLTX-Hh1a and κ_1.3_-TLTX-Ta1a for the toxins of *S. gigantea*, *S. haddoni*, *S. mertensii*, *C. adhaesivum*, *H. hemprichii* and *T. aster*, respectively) have been deposited in the DDBJ/EMBL/GenBank databases under the following accession numbers: AB595204 for κ_1.3_-SHTX-Sg1a (501 bp), AB595205 for κ_1.3_-SHTX-Sha1a (461 bp), AB595206 for κ_1.3_-SHTX-Sm1a (461 bp), AB595207 for κ_1.3_-TLTX-Ca1a (464 bp), AB595208 for κ_1.3_-TLTX-Hh1a (403 bp) and AB595209 for κ_1.3_-TLTX-Ta1a (469 bp). The six cDNAs had the following common features; a stop codon (TAA or TGA) is contained in the 5′-untranslated region upstream of the initiating Met and a poly(A) signal (AATAAA) and a poly(A) tail in the 3′-terminal region. In addition, the cDNAs encoding the toxins of the *Stichodactyla* species and Thalassianthidae species contain open reading frames composed of 222 bp (corresponding to 74 amino acid residues) and 225 bp (corresponding to 75 amino acid residues), respectively. As a typical example, the nucleotide sequence of the κ_1.3_-SHTX-Sg1a cDNA is illustrated in Figure 2 (refer to Supplementary Data for the nucleotide sequences of the cDNAs encoding the remaining five toxins).

### 2.3. Amino acid sequences of type 1 potassium channel toxins

For the precursor proteins of the six type 1 potassium channel toxins (κ_1.3_-SHTX-Sg1a, κ_1.3_-SHTX-Sha1a, κ_1.3_-SHTX-Sm1a, κ_1.3_-TLTX-Ca1a, κ_1.3_-TLTX-Hh1a and κ_1.3_-TLTX-Ta1a) cloned in this study, commonly, the N-terminal segment up to the 21st residue was predicted to be a signal peptide by SignalP analysis [24] (refer to Figure 2 and Supplementary Data). Moreover, the C-terminal segment of 35 residues was judged to be a mature portion based on the known sequences of κ_1.3_-SHTX-Hm1a [15] and κ_1.3_-SHTX-She1a [16]. Thus, the sequence of 18 or 19 residues between the signal peptide and the mature portion should be a propart, which is characterized by ending with a pair of basic residues (Lys-Arg), a cleavage site for subtilisin-like proteases. These structural features are recognized for the precursors of κ_1.3_-SHTX-Hm1a [15] and κ_1.3_-ATTX-Aer1a [12] and also for those of many sea anemone peptide toxins such as δ-ATTX-Aeq2a (Ae I or AeNa) from *Actinia equina* [25], Ω-SHTX-Sg1a (gigantoxin I), δ-SHTX-Sg2a (gigantoxin II) and δ-SHTX-Sg1a (gigantoxin III) from *Stichodactyla gigantea* [26] and U-SHTX-Am1a (Am I), U-SHTX-Am2a (Am II) and δ-SHTX-Am2a (Am III) from *Antheopsis maculata* [27].

**Figure 3:**
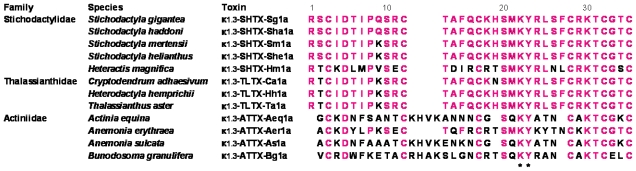
Amino acid sequences of type 1 potassium channel peptide toxins from sea anemones. The residues identical with those of κ_1.3_-SHTX-Sg1a are shown in red. Asterisks under the sequence of κ_1.3_-ATTX-Bg1a represent the dyad (Lys-Tyr) that is crucial for the binding to potassium channels.

The amino acid sequences of the mature portions of the six potassium channel toxins are shown in Figure 3, together with those of the known type 1 potassium channel toxins. Each of the six toxins has six Cys residues in the same positions as the known type 1 toxins, suggesting that the three disulfide bridges are located between ^3^Cys and ^35^Cys, between ^12^Cys and ^28^Cys and between ^17^Cys and ^32^Cys as demonstrated for κ_1.3_-SHTX-Hm1a [15], κ_1.3_-SHTX-She1a [28] and κ_1.3_-ATTX-Bg1a [14]. In addition, the dyad (Lys-Tyr), which is assumed to be crucial for the toxin binding to potassium channels [29–32], is conserved in the six toxins, as in the known type 1 toxins. Interestingly, although the six toxins include three toxins (κ_1.3_-SHTX-Sg1a, κ_1.3_-SHTX-Sha1a and κ_1.3_-SHTX-Sm1a) from *Stichodactyla* species of Stichodactylidae and three toxins (κ_1.3_-TLTX-Ca1a, κ_1.3_-TLTX-Hh1a and κ_1.3_-TLTX-Ta1a) from Thalassianthidae species, they share more than 90% sequence identity with one another and also with κ_1.3_-SHTX-She1a; even the same sequence is seen between κ_1.3_-SHTX-Sg1a and κ_1.3_-SHTX-Sha1a, between κ_1.3_-SHTX-Sm1a and κ_1.3_-SHTX-She1a and between κ_1.3_-TLTX-Hh1a and κ_1.3_-TLTX-Ta1a. In contrast, the six toxins show rather low sequence identities (34–63%) with κ_1.3_-SHTX-Hm1a, κ_1.3_-ATTX-Aeq1a, κ_1.3_-ATTX-Aer1a, κ_1.3_-ATTX-As1a and κ_1.3_-ATTX-Bg1a.

Type 1 potassium channel toxins can be further divided into subtype 1a and 1b toxins, having four and eight amino acid residues, respectively, between the second and third Cys residues from the N-terminus [12], although little is understood as to how the structural difference between these subtypes is related to the potassium channel toxicity. The six toxins cloned in this study are apparently subtype 1a toxins. It is interesting to note that the distribution of subtype 1a and 1b toxins is associated with the taxonomical position of sea anemones. Eight subtype 1a toxins (κ_1.3_-SHTX-Sg1a, κ_1.3_-SHTX-Sha1a, κ_1.3_-SHTX-Sm1a, κ_1.3_-SHTX-She1a, κ_1.3_-SHTX-Hm1a, κ_1.3_-TLTX-Ca1a, κ_1.3_-TLTX-Hh1a and κ_1.3_-TLTX-Ta1a) are contained in members of the family Stichodactylidae or Thalassianthidae and three subtype 1b toxins (κ_1.3_-ATTX-Aeq1a, κ_1.3_-ATTX-As1a and κ_1.3_-ATTX-Bg1a) in those of the family Actiniidae; the only exception is κ_1.3_-ATTX-Aer1a, a subtype 1a toxin in *A. erythraea* (member of the family Actiniidae). At present, no information about the amino acid sequences of subtype 1b toxin precursors is available. In this study, neither κ_1.3_-ATTX-Aeq1a (subtype 1b toxin of *A. equina*) nor a type 1 toxin (presumably subtype 1b toxin) of *M. doreensis* (member of Actiniidae) could be cloned, since no amplified products were obtained by RT-PCR using the degenerate primers (RT-f and RT-r). Therefore, molecular cloning of κ_1.3_-ATTX-Aeq1a using degenerate primers designed from its amino acid sequence is now under study.

It is apparent that the six type 1 potassium channel toxins (κ_1.3_-SHTX-Sg1a, κ_1.3_-SHTX-Sha1a, κ_1.3_-SHTX-Sm1a, κ_1.3_-TLTX-Ca1a, κ_1.3_-TLTX-Hh1a and κ_1.3_-TLTX-Ta1a) are individually expressed in the six species of sea anemones, since their cDNAs were cloned using total RNA as the starting material. However, the potassium channel toxicity detected in each sea anemone by competitive inhibition experiments using ^125^I-α-dendrotoxin may not be explained only by the type 1 toxin cloned. Indeed, one type 2 toxin (κ_1.3_-SHTX-Sha2a) and two type 4 toxins (κ_1.3_-SHTX-Sha3a and b) had been found in *S. haddoni* [17] from which κ_1.3_-SHTX-Sha1a was cloned in this study. Future study is needed to isolate and characterize potassium channel toxins from the positive species. Such study might discover structurally and functionally novel potassium channel toxins.

## 3. Experimental Section

### 3.1. Sea anemone samples

The following 23 species of sea anemones belonging to seven families were used in this study: *Actinia equina*, *Cnidopus japonicus*, *Macrodactyla doreensis*, *Telactinia citrina*, *Boloceroides mcmurrichi*, *Calliactis japonica*, *Calliactis polypus*, *Telmatactis clavata*, *Phymanthus loligo*, *Antheopsis koseirensis*, *Antheopsis maculata*, *Entacmaea ramsayi*, *Heteractis* (*Radianthus*) *aurora*, *Heteractis* (*Radianthus*) *gelam*, *Heteractis* (*Radianthus*) *magnifica*, *Mesactinia ganensis*, *Stichodactyla gigantea, Stichodactyla haddoni*, *Stichodactyla mertensii*, *Stichodactyla tapetum*, *Cryptodendrum adhaesivum*, *Heterodactyla hemprichii* and *Thalassianthus aster* (refer to Table 2 for their taxonomical position). Except for two species (*A. equina* and *S. gigantea* used only for cDNA cloning), 21 species were used for screening of Kv1 potassium channel toxins. Specimens of *A. equina* were collected at Katsuura, Chiba Prefecture; those of *B. mcmurrichi* and *A. maculata* at Shishijima, Kagoshima Prefecture; those of *T. clavata* at Okinoshima, Chiba Prefecture; and those of *C. polypus* along the coast of Ishigaki Island, Okinawa Prefecture. These specimens were transported frozen or alive to our laboratory. For the remaining species, live specimens were purchased from retail aquarium shops. Most of the specimens were stored at −20 °C until extraction. For cDNA cloning, one live specimen of each species was cut into small pieces, which were immediately frozen in liquid nitrogen and stored at −80 °C until use.

### 3.2. Preparation of crude extracts

Each frozen sample was well macerated and 2–3 g of the macerate was homogenized in five volumes of distilled water. After centrifugation, the supernatant obtained was used as a crude extract.

### 3.3. Assay of potassium channel toxicity

Potassium channel toxicity was indirectly assayed by competitive inhibition of the binding of ^125^I-α-dendrotoxin to rat synaptosomal membranes, as reported previously [11,33]. Synaptosomal membrane suspension (0.4 mg protein/mL) was prepared from rat brains (Funakoshi, Tokyo, Japan). Labeling of α-dendrotoxin (Sigma, St. Louis, MO, U.S.) with ^125^I was performed by the chloramine-T (*N*-chloro-p-toluenesulphonamide) method according to the instructions of GE-Healthcare Biosciences and ^125^I-α-dendrotoxin (3.52 TBq/mmol) was purified by gel filtration on a Sephadex G-10 column (1.2 × 2.5 cm; GE-Healthcare Biosciences). For competitive binding experiments, the reaction mixture (200 μL of the synaptosomal membrane suspension, 40 μL of sample solution and 10 μL of 606 pM ^125^I-α-dendrotoxin) was incubated at room temperature for 30 min. The membranes were then collected by centrifugation and measured for radioactivity on a COBRA II gamma counter (Packard, Meriden, CT, U.S.). The highest binding (100% binding) was known by replacing sample solution with distilled water. On the other hand, non-specific binding (about 20%) was determined by replacing sample solution with 1.42 μM non-labeled α-dendrotoxin and subtracted from each datum.

### 3.4. Cloning experiments

Molecular cloning of type 1 potassium channel toxins (κ_1.3_-SHTX-Sg1a of *S. gigantea*, κ_1.3_-SHTX-Sha1a of *S. haddoni*, κ_1.3_-SHTX-Sm1a of *S. mertensii*, κ_1.3_-TLTX-Ca1a of *C. adhaesivum*, κ_1.3_-TLTX-Hh1a of *H. hemprichii* and κ_1.3_-TLTX-Ta1a of *T. aster*) was performed by a combination of RT-PCR, 3′RACE and 5′RACE. Designations and nucleotide sequences of the primers used in this study are summarized in Table 2. PCR amplifications were all carried out using Ex Taq DNA polymerase (Takara Bio, Otsu, Japan) under the following conditions: pre-incubation at 98 °C for 2 min; 30 cycles consisting of denaturation at 98 °C for 10 s, annealing at 53 °C for 30 s and extension at 72 °C for 3 min; and final extension at 72 °C for 7 min. Amplified products were subcloned into the pT7Blue T-vector (Novagen, Darmstadt, Germany) and nucleotide sequences were determined using a Cy5 ThermoSequence Dye Terminator Kit (GE-Healthcare Biosciences) and a Long-Read Tower DNA Sequencer (GE-Healthcare Biosciences).

Total RNA was extracted from 1 g of the frozen sample with TRIzol reagent (Invitrogen, Carlsbad, CA, U.S.). From 5 μg of total RNA, cDNA was synthesized using a 3′RACE System for Rapid Amplification of cDNA Ends Kit (Invitrogen), as recommended by the manufacturer, and used as a template in RT-PCR and 3′RACE. Of the known type 1 potassium channel toxins, only two toxins, κ_1.3_-ATTX-Aer1a from *A. erythraea* [12] and κ_1.3_-SHTX-Hm1a from *Heteractis magnifica* [15], have so far been cloned for their cDNAs. For RT-PCR, therefore, a pair of degenerate primers (RT-f and RT-r) were designed from the highly conserved regions of κ_1.3_-ATTX-Aer1a and κ_1.3_-SHTX-Hm1a; RT-f corresponded to the segment LIALCMS (κ_1.3_-ATTX-Aer1a) or LIAFCLC (κ_1.3_-SHTX-Hm1a) in the signal peptide and RT-r to the segment CRTSMKYK (κ_1.3_-ATTX-Aer1a) or CRTSMKYR (κ_1.3_-SHTX-Hm1a) in the mature peptide (refer to Figure 3). Based on the results obtained by RT-PCR, 3′-terminal sequences were analyzed by 3′RACE, in which a gene-specific primer (3′-Sha-f for κ_1.3_-SHTX-Sha1a, 3′-Ca-f for κ_1.3_-TLTX-Ca1a or 3′-Hh-f for κ_1.3_-TLTX-Hh1a) was used in combination with the abridged universal amplification primer (AUAP). Then, the remaining 5′-terminal sequences were analyzed by 5′RACE as follows. From 5 μg of total RNA, cDNA as a template was synthesized using a 5′RACE System for Rapid Amplification of cDNA Ends Kit (Invitrogen) and a gene-specific primer (5′-Syn-SgSha-r for κ_1.3_-SHTX-Sg1a and κ_1.3_-SHTX-Sha1a, 5′-Syn-Sm-r for κ_1.3_-SHTX-Sm1a or 5′-Syn-CaHhTa-r for κ_1.3_-TLTX-Ca1a, κ_1.3_-TLTX-Hh1a and κ_1.3_-TLTX-Ta1a). The first 5′RACE reaction was completed using a gene-specific primer (5′-Sg-r for κ_1.3_-SHTX-Sg1a, 5′-ShaSm-r for κ_1.3_-SHTX-Sha1a and κ_1.3_-SHTX-Sm1a, 5′-Ca-r for κ_1.3_-TLTX-Ca1a or 5′-HhTa-r for κ_1.3_-TLTX-Hh1a and κ_1.3_-TLTX-Ta1a) and the abridged anchor primer (AAP). In the case of κ_1.3_-SHTX-Sha1a and κ_1.3_-SHTX-Sm1a, nested PCR was further performed using a gene-specific primer (5′-Sg-r for κ_1.3_-SHTX-Sha1a and κ_1.3_-SHTX-Sm1a) and AUAP.

## 4. Conclusions

Kv1 potassium channel toxins are widely distributed in sea anemones, including not only members of the two families (Actiniidae and Stichodactylidae) but also of other families such as Thalassianthidae. Based on the amino acid sequence features, type 1 potassium channel toxins are divided into two subtypes (subtypes 1a and 1b). Subtype 1a toxins are contained in members of Stichodactylidae and Thalassianthidae and subtype 1b toxins in Actiniidae; the only exception is κ_1.3_-ATTX-Aer1a, a subtype 1a toxin in *Anemonia erythraea* (member of Actiniidae).

## Supplementary Data



## Figures and Tables

**Table 1:** Distribution of Kv1 potassium channel toxins in sea anemones.

Sea anemone			Potassium channel toxin	Reference
Suborder	Family	Species		
Endocoelantheae	Halcuriidae	*Halcurias* sp.	−	[22]
Nynantheae	Actiniidae	*Actinia bermudensis*	+	[21]
		*Actinia equina*	+	[11,22]
		*Adamsia maculata*	−	[21]
		*Alcyonium digitatum*	−	[21]
		*Anemonia erythraea*	+	[12,22]
		*Anemonia sulcata* (*viridis*)	+	[13,21]
		*Anthopleura asiatica*	−	[22]
		*Anthopleura japonica*	−	[22]
		*Anthopleura pacifica*	−	[22]
		*Anthopleura xanthogrammica*	−	[22]
		*Bunodosoma cangicum*	+	[21]
		*Bunodosoma granulifera*	+	[14,21]
		*Cnidopus japonicus*	−	This study
		*Condylactis passiflora*	−	[22]
		*Dofleinia armata*	−	[22]
		*Macrodactyla doreensis*	+	This study
		*Tealia coriacea*	−	[22]
		*Telactinia citrina*	+	This study
	Actinodendronidae	*Actinodendron plumosum (arboreum*)	−	[22]
	Boloceroididae	*Boloceroides mcmurrichi*	−	This study
	Diadumenidae	*Haliplanella lineata*	−	[22]
	Hormathiidae	*Calliactis japonica*	−	This study
		*Calliactis polypus*	+	This study
	Isophellidae	*Telmatactis clavata*	−	This study
	Metridiidae	*Metridium senile*	−	[22]
	Nemanthidae	*Nemanthus nitidus*	−	[22]
	Phymanthidae	*Phymanthus loligo*	−	This study
	Stichodactylidae	*Antheopsis koseirensis*	−	This study
		*Antheopsis maculata*	−	This study
		*Entacmaea actinostoloides*	−	[22]
		*Entacmaea ramsayi*	−	This study
		*Heteractis* (*Radianthus*) *aurora*	−	This study
		*Heteractis* (*Radianthus*) *crispus*	−	[22]
		*Heteractis* (*Radianthus*) *gelam*	−	This study
		*Heteractis* (*Radianthus*) *magnifica*	+	[15], This study
		*Mesactinia ganensis*	+	This study
		*Stichodactyla gigantea*	+	This study a
		*Stichodactyla haddoni*	+	[17], This study
		*Stichodactyla helianthus*	+	[16,21]
		*Stichodactyla mertensii*	+	[21], This study
		*Stichodactyla tapetum*	+	This study
	Thalassianthidae	*Cryptodendrum adhaesivum*	+	This study
		*Heterodactyla hemprichii*	+	This study
		*Thalassianthus aster*	+	This study

a The occurrence of potassium channel toxins was established by cDNA cloning, not by screening.

**Table 2:** Designations and nucleotide sequences of the primers used in this study.

Experiment	Designation of primer	Nucleotide sequence of primer
RT-PCR	RT-f	5′-GATCGCAYTTTGTMTGTSTGT-3′
	RT-r	5′-TGTATTTCATTGACGTYCTACA-3′
3′RACE	3′-Sha-f	5′-ACGGTTTCCAAAAGAGGAGG-3′
	3′-Ca-f	5′-GAACTGCAGGATGTCGAGGA-3′
	3′-Hh-f	5′-GAATTGCAGGATGACGAGGA-3′
	AUAP	5′-GGCCACGCGTCGACTAGTAC-3′
5′RACE	5′-Syn-SgSha-r	5′-ACAAAAGCTCAGTCTGTATTTC-3′
	5′-Syn-Sm-r	5′-ACAGAAGCTAAGTCTGTATTTC-3′
	5′-Syn-CaHhTa-r	5′-TTGAAATGCTGTGCATCGGC-3′
	5′-Sg-r	5′-TCCTGCAGTTCCATCCTGG-3′
	5′-ShaSm-r	5′-CCTCCTCTTTTGGAAACCGT-3′
	5′-Ca-r	5′-GTCCTCCTCTTTTGGAAAGA-3′
	5′-HhTa-r	5′-GTGCATCGGCTCTTCGGTA-3′
	AUAP	5′-GGCCACGCGTCGACTAGTAC-3′
	AAP	5′-GGCCACGCGTCGACTAGTACGGGGGGGGGGGGGGGG-3′
