# Supplementary figures and images for: Screening and cDNA Cloning of Kv1 Potassium Channel Toxins in Sea Anemones
Source: Mar Drugs. 2010 Dec 2;8(12):2893–905. doi: 10.3390/md8122893 (PMC3039155; doi:10.3390/md8122893)

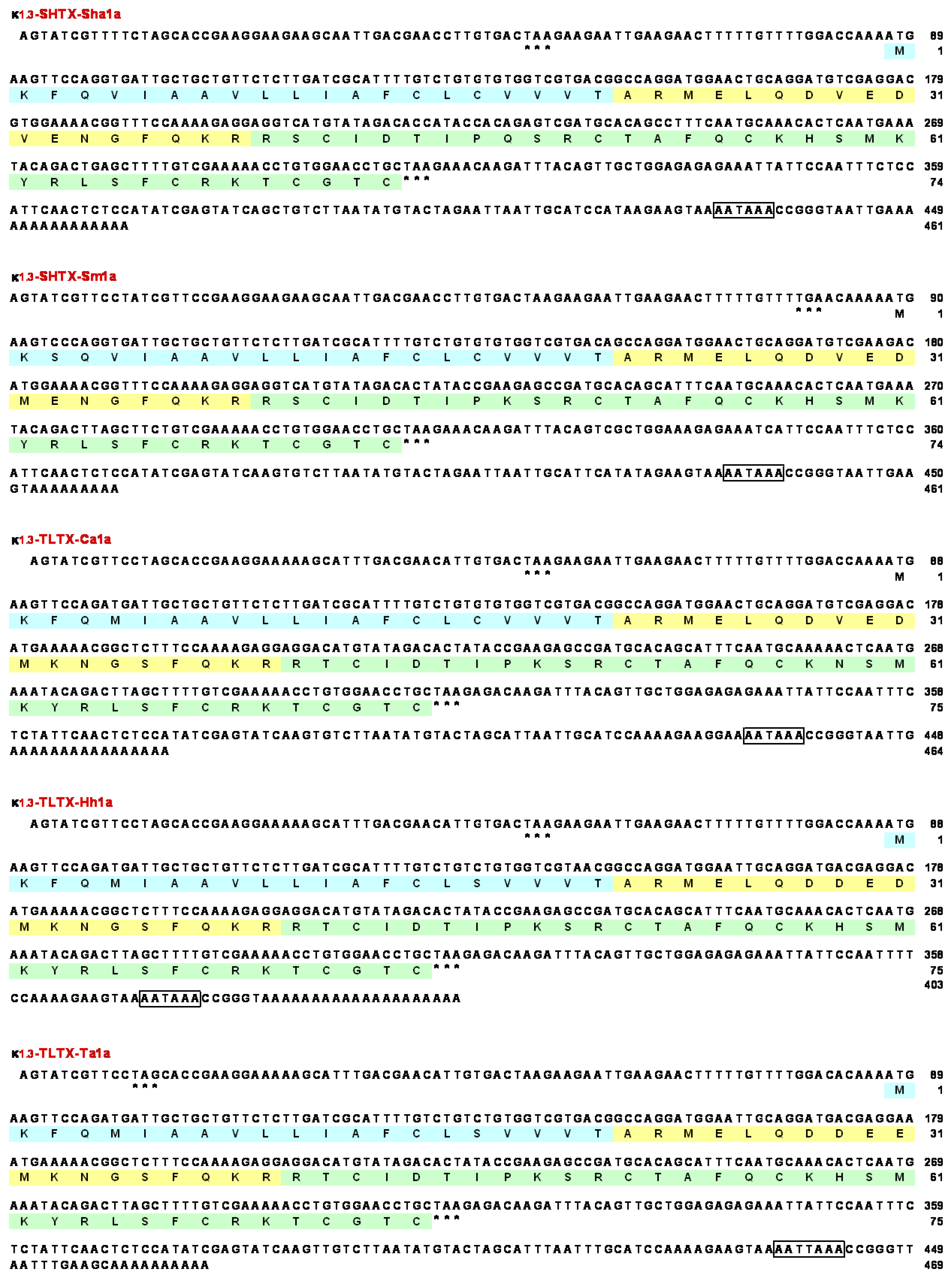

Supplement: Supplementary file 1 [file marinedrugs-08-02893s1.tif]
